# The complete chloroplast genome sequence of *Camellia tachangensis* F. C. Zhang (Theaceae)

**DOI:** 10.1080/23802359.2019.1673247

**Published:** 2019-10-04

**Authors:** Wan-Jun Hao, Jian-Qiang Ma, Chunlei Ma, Jiqiang Jin, Liang Chen

**Affiliations:** Key Laboratory of Tea Biology and Resources Utilization, Ministry of Agriculture and Rural Affairs, Tea Research Institute of the Chinese Academy of Agricultural Sciences, Hangzhou, Zhejiang, China

**Keywords:** Theaceae, *Camellia tachangensis*, chloroplast genome, genetic evolution

## Abstract

To understand genetic background and phylogenetic position of *Camellia tachangensis*, we determined its complete chloroplast genome sequence which is 157,026 bp in length with overall GC content of 36.7%. It has four sub regions: a large single-copy (LSC) region (86,669 bp) and a small single-copy (SSC) region (18,253 bp) are separated by two inverted repeat (IR) regions (26,052 bp each). A total of 129 genes were annotated, containing 86 protein-coding genes, 35 tRNA genes, and 8 rRNA genes. Phylogenetic trees showed *C. tachangensis* clustered with *Camellia gymnogyna* and *Camellia taliensis* and separated from *Camellia sinensis* and its two varieties, *Camellia sinensis* var. *assamica* and *Camellia sinensis* var. *pubilimba.*

*Camellia* is the economically most important genus in the family Theaceae. It contains more than 200 species and is regarded as one of the most difficult taxa in plants (Vijayan et al. 2009). Until now, taxonomists still have different opinions on the taxa of section *Thea* of the genus *Camellia.* The chloroplast genome sequences could provide valuable information for taxonomic classification and the reconstruction of phylogeny for resolving complex evolutionary relationships (Jansen et al. 2007; Parks et al. 2009). In the past few years, the complete chloroplast genome sequences of most species in section *Thea* were determined (Yang et al. 2013; Chen et al. 2014; Huang et al. 2014; Dong et al. 2018; Li et al. 2018; Zeng et al. 2018). However, the complete chloroplast genome sequence of *Camellia tachangensis* F. C. Zhang has not been reported. In this study, we characterized the complete chloroplast genome sequence of *C. tachangensis* as a resource for better understanding the diversification and evolution of the genus *Camellia*.

The young leaves were collected from *C. tachangensis* cultivar Xingyi6, growing in the China National Germplasm Hangzhou Tea Repository (30.179584N, 120.093516E). A voucher specimen (GS00125) was deposited in the Herbarium of the Germplasm Resources Laboratory of the Tea Research Institute of the Chinese Academy of Agricultural Sciences. The DNA sequencing was performed on an Illumina Hiseq 2500 platform (Illumina, San Diego, CA). The chloroplast genome was *de novo* assembled using NOVOPlasty (Dierckxsens et al. 2017) with the *rbcL* gene sequence (Genbank: MH270471.1) as seed and annotated using GeSeq (Tillich et al. 2017).

The complete chloroplast genome of ‘Xingyi6’ (Genbank: MN327576) is 157,026 bp in length with the overall GC ratio of 36.7%. It composed of a large single-copy (LSC) region of 86,669 bp and a small single-copy (SSC) region of 18,253 bp separated by two inverted repeat (IR) regions of 26,052 bp each. A total of 129 genes were annotated, containing 86 protein-coding genes, 35 tRNA genes, and 8 rRNA genes. Among them, four rRNA genes (i.e. *4.5S*, *5S*, *16S*, and *23S rRNA*), seven protein-coding genes (i.e. *ndhB*, *rpl2*, *rpl23*, *rps12*, *rps7*, *ycf15*, *ycf1*, and *ycf2*), and seven tRNA genes (i.e. *trnM*, *trnL*, *trnV*, *trnE*, *trnA*, *trnR*, and *trnN*) occur in double copies in IR regions. There are two protein-coding genes (*clpP* and *rps12*) containing two introns and 11 protein-coding genes (*atpF*, *ndhA*, *ndhB × 2*, *petB*, *petD*, *rpl2 × 2*, *ropC1*, *rps16*, and *ycf3*) containing one intron.

Using clustalw2.1, we aligned eight chloroplast genomes of different species from the genus *Camellia*. A molecular phylogenetic tree was constructed with the MEGA X (Kumar et al. 2018) using neighbour-joining (NJ) method with 10,000 bootstrap replicates (Figure 1). The result showed *C. tachangensis*, *Camellia gymnogyna*, and *Camellia taliensis* were clustered into a group. *Camellia sinensis* and its two varieties, *Camellia sinensis* var. *assamica* and *Camellia sinensis* var. *pubilimba* were clustered into another group. This chloroplast genome will provide valuable information for genetic evolution studies of *Camellia*.

**Figure 1.**
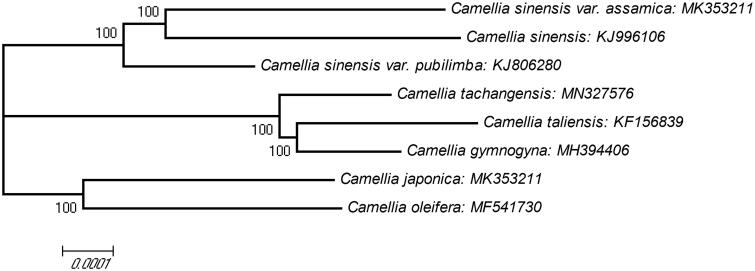
The phylogenetic tree based on eight complete chloroplast genome sequences of different species from the genus *Camellia*. The phylogenetic tree was constructed using neighbour-joining (NJ) method with 10,000 bootstrap replicates. The bootstrap values are labelled at each branch nodes.
